# Molecular detection of *Coxiella burnetii* and *Coxiella* species in rats and chickens from poultry farms in North West Province, South Africa

**DOI:** 10.1002/vms3.1192

**Published:** 2023-08-17

**Authors:** Tsepo Ramatla, Zamantungwa T. H. Khumalo, Asiashu Matshotshi, Kgaugelo E. Lekota, Moeti O. Taioe, Oriel Thekisoe

**Affiliations:** ^1^ Unit for Environmental Sciences and Management North‐West University Potchefstroom South Africa; ^2^ Vectors and Vector‐borne Diseases Research Programme, Department of Veterinary Tropical Diseases University of Pretoria Onderstepoort South Africa; ^3^ Study Operations Clinvet International Bloemfontein South Africa; ^4^ Department of Life and Consumer Sciences University of South Africa Florida South Africa; ^5^ Epidemiology, Parasites and Vectors Agricultural Research Council, Onderstepoort Veterinary Research Onderstepoort South Africa

**Keywords:** chickens, *Coxiella burnetii*, Q‐fever, *Rattus* species, South Africa

## Abstract

**Background:**

*Coxiella burnetii* is a bacterial pathogen that causes query fever and coxiellosis in humans and animals, respectively. There is a scarcity of studies on the prevalence of *C. burnetii* infections in rats and chickens in South Africa.

**Objective:**

The aim of this study was to determine the occurrence of *C. burnetii* in rats and chickens sampled from poultry farms in the North West Province of South Africa.

**Methods:**

DNA was extracted from rodent kidneys (*n* = 68) and chicken faeces (*n* = 52). Two rodent pest species, namely *Rattus rattus* and *Rattus tanezumi*, were identified by analysis of *CO1* gene sequences. Detection of *C. burnetii* was carried out using polymerase chain reaction assays targeting *23S rRNA*, *16S rRNA* and *IS111* markers.

**Results:**

*C. burnetii* was detected in 16.2%, 8.8% and 25% of *R. rattus*, *R. tanezumi* and chickens, respectively.

**Conclusions:**

The findings in this study demonstrate that rodents and chickens are harbouring *C. burnetii* at sampled poultry farms. There should be frequent screening for *C. burnetii* in poultry operations. The likelihood of future transmission between rodents and chickens, including humans, also needs to be investigated.

## Graphical abstract

DNA was extracted from rodent kidneys (*n* = 68) and chicken faeces (*n* = 52). Two rodent pest species, namely *Rattus rattus* and *Rattus tanezumi*, were identified by the analysis of *CO1* gene sequences. Detection of *Coxiella burnetii* was carried out using polymerase chain reaction (PCR) assays targeting *23S rRNA*, *16S rRNA* and *IS111* markers. *C. burnetii* was detected in 16.2%, 8.8% and 25% of *R. rattus*, *R. tanezumi* and chickens, respectively.

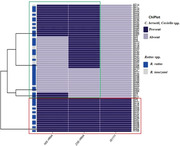

## 1 INTRODUCTION

Query fever (Q‐fever) is a zoonotic disease caused by an obligate intercellular bacteria *Coxiella burnetii* in humans (Mangena et al., 2021, Sethi et al., 1978), which also causes coxiellosis in animals (Cabrera Orrego et al., 2020). During desiccation or sunlight, *C. burnetii* develops spore‐like forms that resist environmental stressors. As a result, the bacteria can survive for long periods of time under adverse conditions in soil or other dry materials (Evstigneeva et al., 2007; Körner et al., 2021). *C. burnetii* is a strictly intracellular, Gram‐negative bacterium that is distributed worldwide except for New Zealand (Maurin & Raoult, 1999). Similarly, *Coxiella*‐like bacteria are a group of bacteria that remain to be isolated and are characterized as phylogenetically close to *C. burnetii* (Rahal et al., 2020). Duron et al. (2015) published a recent study in which all *C. burnetii* strains were shown to descend from a *Coxiella*‐like progenitor. It was, however, established that these strains pose a much lower infection risk to vertebrates than *C. burnetii* (Duron et al., 2015).

Domestic ruminants, including sheep, goats and cattle, are thought to be the primary source of human infection outbreaks (Cabrera Orrego et al., 2020; Tawana et al., 2022). Other hosts, such as birds and ticks, are natural reservoirs of *C. burnetii* (Maurin & Raoult, 1999). Additionally, rodents contribute to the transmission of *C. burnetii* (Thompson et al., 2012).

Recently, Q‐fever has gained attention due to a number of outbreaks in different countries, the majority of which have been linked to domestic animals (Simpson et al., 2018; Sprong et al., 2012). Farmers, veterinarians, abattoir workers and laboratory personnel culturing *C. burnetii* and, more importantly, working with *C. burnetii*–infected animals are all at risk of falling sick with Q‐fever illness (Frean, 2022; Maurin & Raoult, 1999; Mioni et al., 2022). The first recorded cases of human Q‐fever in South Africa were documented in 1950 (Gear et al., 1950). The endemic status of Q‐fever in South Africa is not widely known, and this could be associated with the fact that the Animal Diseases Act 35 of 1984 does not recognize the illness as a controlled or a notifiable disease (Maurin & Raoult, 1999). Serological survey of Q‐fever comprised few studies carried out in South Africa (De Boni et al., 2022; Donnelly et al., 2021; Gummow et al., 1987; Mangena et al., 2021).

There is a lack of data regarding molecular epidemiological studies of *C. burnetii* occurrence in rats and chickens in South Africa. The main aim of this study was to investigate the occurrence of *C. burnetii* infections in rat and chicken samples obtained from poultry farms in South Africa's North West Province.

## 2 MATERIALS AND METHODS

### 2.1 Sample collection and DNA extraction

**FIGURE 1:**
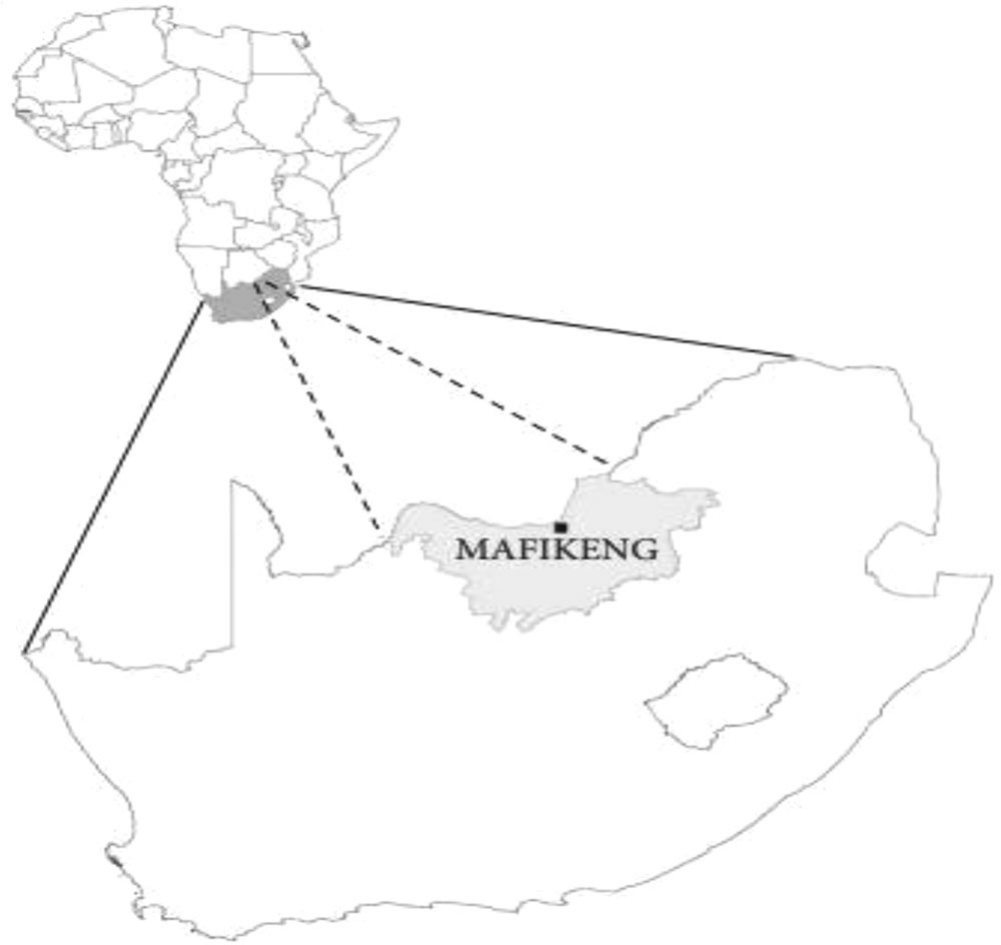
Map of Africa showing the Mahikeng sampling area in the North West Province of South Africa. *Source*: This figure is adapted from a version published previously (Ramatla et al., 2019).

Samples were collected from chickens (*n* = 52) and rats (*n* = 68) at six commercial farms in Ngaka Modiri Molema District (26.0282°S, 25.8522°E) in Mahikeng Local Municipality of the North West Province, South Africa (Figure 1). Wild rats were captured within poultry farm enclosures and euthanized as previously described by Ramatla et al. (2019). Tissue samples were harvested from the rodent kidneys. Fresh chicken faecal samples were collected in chicken enclosures within poultry houses. Genomic DNA was extracted from both rat tissues and chicken faeces using the DNeasy Blood & Tissue Kit (Qiagen, Germany) following manufacturer's instructions.

### 2.2 Polymerase chain reaction (PCR) and sequencing

Polymerase chain reaction (PCR) and sequencing of the *Cytochrome c oxidase I* (*CO1*) gene for rodent identification were carried out as described previously (Ramatla et al., 2019). A nested PCR (nPCR) was used to amplify the *16S* rRNA and *23S* rRNA gene markers of *Coxiella* spp. First‐round PCR was performed using the primers Cox‐23SF1 (5′‐GCC TGC GAW AAG CTT CGG GGA G‐3′) and Cox‐23SR2 (5′‐CTC CTA KCC ACA SCT CAT CCC C‐3′) which produced 694–1188 bp amplicons. The second round of PCR used Cox‐23SF2 (5′‐GAT CCG GAG ATW TCY GAA TGG GG‐3′) and Cox‐23SR1 (5′‐TCGYTCGGTTTCGGGTCKACTC‐3′) primers, which yielded 583–867 bp (Mofokeng et al., 2022). For the *16S* rRNA gene marker, first‐round PCR was performed with the primers Cox16SF1 (5′‐CGT AGG AAT CTA CCT TRT AGW GG‐3′) and Cox16SR2 (5′‐GCC TAC CCG CTT CTG GTA CAA TT‐3′) yielding 1321–1429 bp amplicons. Then, a second round of PCR was performed with primers Cox16SF2 (5′‐TGA GAA CTA GCT GTT GGR RAG T‐3′) and Cox16SR2, yielding 624–627 bp amplicons (Seo et al., 2016). Each PCR reaction included a total reaction volume of 25 μL containing 12.5 μL of a 2× DreamTaq Green Master Mix (0.4 mM dATP, 0.4 mM dCTP, 0.4 mM dGTP, 0.4 mM dTTP, 4 mM MgCl_2_ and loading buffer) (Thermo Fisher Scientific, South Africa), 8.5 μL of nuclease‐free water, 2.0 μL of the template DNA and 1.0 μL of each oligonucleotide primer. Pre‐denaturation at 93°C for 3 min; 30 cycles of denaturation at 93°C for 30 s, annealing at 56°C for 30 s, and polymerization at 72°C for 1 min; and a final elongation step at 72°C for 5 min. The PCR reactions were carried out on an Applied Biosystems ProFlex PCR System (Applied Biosystems, Singapore).

### 2.3 Identification of *Coxiella burnetii* using the *IS1111* gene

To further identify this bacterium, a species‐specific conventional PCR targeting the *IS1111* gene of *C. burnetii* was conducted with IS1 F (5′‐CGC AGC ACG TCA AAC CG‐3′) and IS1R (5′‐TAT CTT TAA CAG CGC TTG AAC GTC‐3′) primers, yielding 146 bp amplicons (De Bruin et al., 2011). The following PCR conditions were used: pre‐denaturation for 15 min at 95°C; 35 cycles of denaturation for 30 s at 95°C, annealing for 30 s at 60°C and polymerization for 1 min at 72°C; and a final elongation step for 10 min at 72°C. The PCR products were resolved on a 1.5% (w/v) agarose gel stained with 0.001 g/mL ethidium bromide and visualized under ultraviolet illumination using the ENDURO (Labnet International Inc., USA).

### 2.4 Statistical analysis

Data from the samples that tested positive for different PCR detection methods were analysed on Microsoft Excel Professional 2016 (Analysis Tool Package). Positive samples were summarized as percentages and tested at 95% confidence intervals of the mean. Cohen's weighted kappa (*κ*) was used to measure rates of agreement between samples that was positive across different farms with the *α* value that was set at 0.05, whereby *κ* values ≤0 as indicating no agreement and 0.01–0.20 as none to slight, 0.21–0.40 as fair, 0.41–0.60 as moderate, 0.61–0.80 as substantial and 0.81–1.00 as almost perfect agreement (McHugh, 2012). Heatmap analyses of *23S* rRNA, *16S* rRNA and *IS111* markers for distinguishing between *C. burnetii* and *Coxiella*‐Like species from *Rattus* spp. and chickens were constructed using ChiPlot with default settings (http://chiplot.online/).

## 3 RESULTS

### 3.1 Rodent identification

Based on the *CO1* gene analysis described in our previous study, a total of *n* = 68 collected rats were identified as *Rattus rattus* (69.1%) and *Rattus tanezumi* (30.9%) species (Ramatla et al., 2019).

### 3.2 Detection of *Coxiella burnetii*

The *23S* rRNA PCR detected *C. burnetii* infections of 65.4% (*n* = 34), 32.7% (*n* = 17) and 64.8% (*n* = 54) in *R. rattus*, *R. tanezumi* and chicken samples, respectively. Detailed PCR results of *C. burnetii* from different farms are presented in Table 1.

**TABLE 1:** Number of *Coxiella burnetii* positive samples from rat and chicken samples at six different farms.

Farm	Source of sample	Total number of samples	*23S rRNA* (%)	*16S rRNA* (%)	*IS111 gene* (%)	Cohen's weighted kappa (*κ*)
**A**	*Rattus Rattus*	8	4 (50)	3 (37.5)	4 (50)	−0.09091
	*Rattus tanezumi*	4	3 (75)	3 (75)	1 (25)	
	Chickens	9	8 (88.9)	7 (77.8)	3 (33.3)	
**B**	*R. rattus*	3	3 (100)	3 (100)	2 (66.7)	−0.22777
	*R. tanezumi*	0	0	0	0	
	Chickens	8	8 (100)	5 (62.5)	2 (25)	
**C**	*R. rattus*	11	7 (63.6)	6 (54.5)	1 (9)	0.091073
	*R. tanezumi*	4	2 (50)	2 (50)	2 (50)	
	Chickens	9	6 (66.7)	4 (44.4)	1 (11.1)	
**D**	*R. rattus*	10	9 (90)	7 (70)	3 (30)	0.129485
	*R. tanezumi*	5	4 (80)	2 (40)	1 (20)	
	Chickens	9	4 (44.4)	5 (55.5)	1 (11.1)	
**E**	*R. rattus*	11	8 (72.7)	6 (54.5)	2 (18.2)	0.22222
	*R. tanezumi*	7	7 (100)	3 (42.9)	0	
	Chickens	9	3 (33.3)	5 (55.6)	2 (22.2)	
**F**	*R. rattus*	4	4 (100)	0	0	0.110588
	*R. tanezumi*	1	1 (100)	1 (100)	1 (100)	
	Chickens	8	6 (75)	7 (87.5)	4 (50)	

*Note*: From this table, based on the interpretation of the kappa value, there is no agreement in the observation of the samples that are positive for farms A and B. Furthermore, there is a slight agreement in observations of samples that tested positive for *Coxiella* from farms C, D and F whereas there is a fair agreement in observations from farm E.

The BLASTn results of the *23S* rRNA nucleotide sequences of *Coxiella* spp. detected in this study (GenBank accession numbers: ON872212, ON872213, ON872214 and ON872215) were similar to *Coxiella* spp. sequences on the NCBI database (GenBank accession numbers: ON045549.1 [USA], X79704.1 [Zambia] and NR131209.1 [USA]) with matching pairwise identity scores ranging between 97% and 100%.

A total of 36 *Rattus* spp. tested positive for *Coxiella* spp. infection using the *16S rRNA* PCR. All the samples that were positive for either the *23S rRNA* or *16S rRNA* marker were considered to be other *Coxiella* spp. A total of 25 *Coxiella* spp. infections were positively detected in *R. rattus*, whereas 11 and 33 were detected in *R. tanezumi* and chickens, respectively. The BLASTn search results of the *16S rRNA* indicated that *Coxiella* spp. sequences of this study (GenBank accession numbers: OP688473, OP688474, OP688475, OP688476, OP688477, OP688478 and OP688479) were similar to other *Coxiella* spp. sequences available on the NCBI database (GenBank accession numbers: NR131209.1 [USA] and X79704.1 [Zambia]). This resulted in nucleotide identities ranging from 97% to 99%.

The *IS111* species‐specific PCR detected higher *C. burnetii* infections in *R. rattus* at 16.2% (11/68) as compared to 8.8% (6/68) in *R. tanezumi* host. *C. burnetii* was also detected in 25% (13/52) of chicken samples. The BLASTn search results for the *IS111* gene indicated that the *C. burnetii* sequences of this study (GenBank accession numbers: ON994580, ON994581 and ON994582) Matched with relevant *C. burnetii* sequences available in GenBank (GenBank accession numbers: MT268532.1 [Algeria], CP115461.1 [USA] and CP103428.1 [France]) with identities ranging from 100%.

**FIGURE 2:**
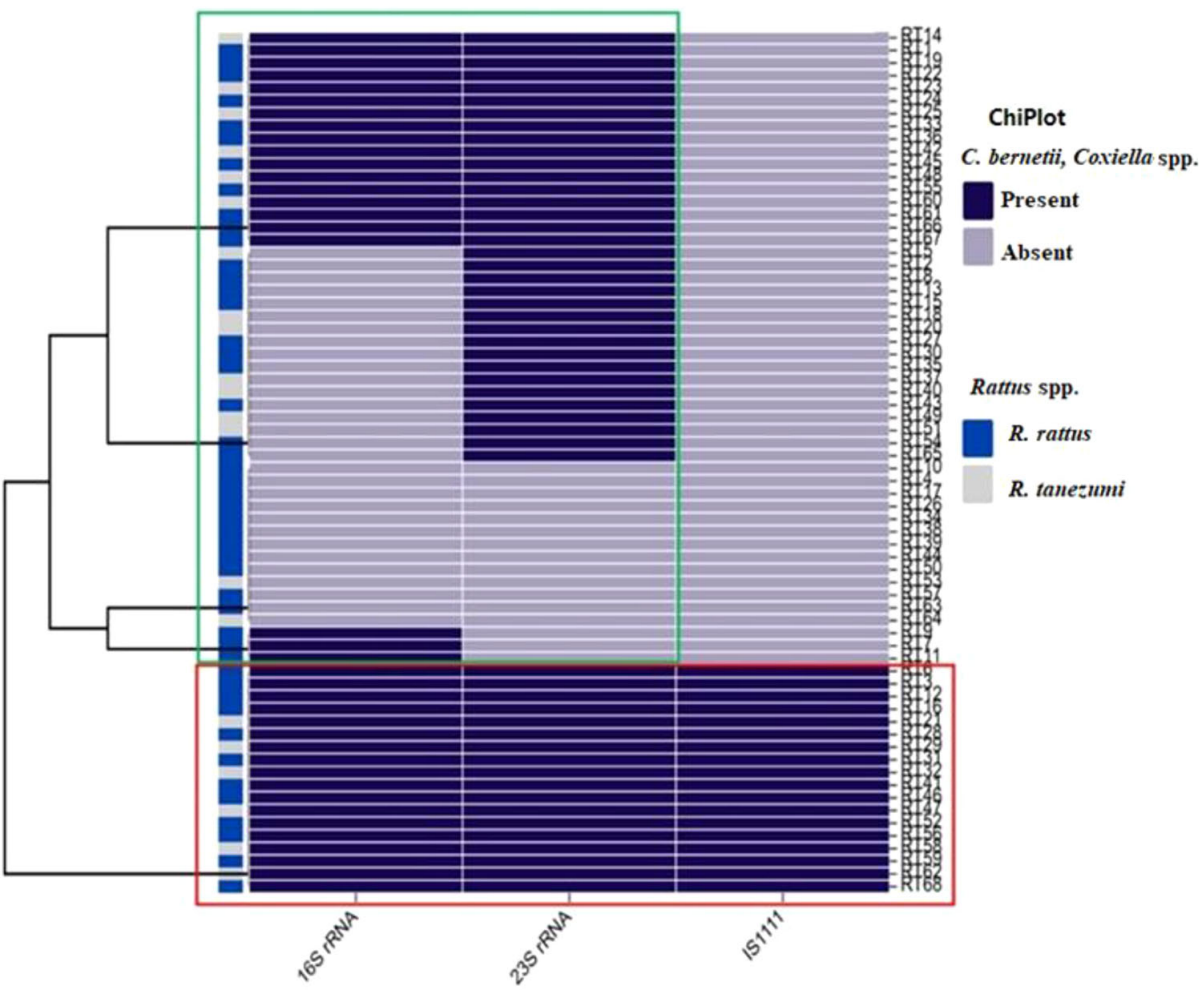
Heatmap showing analyses of *23S rRNA*, *16S rRNA* and *IS111* markers for distinguishing between *Coxiella burnetii* and *Coxiella* spp. from *Rattus* spp. The dark blue colour represents the presence of *Coxiella* spp., grey absence of *Coxiella* spp., whereas the light blue colour represents the presence of *Rattus rattus* and the light grey represents the presence of *Rattus tanezumi*. Samples within the red box are confirmed as *C. burnetii*, whereas green box as *Coxiella* spp.

**FIGURE 3:**
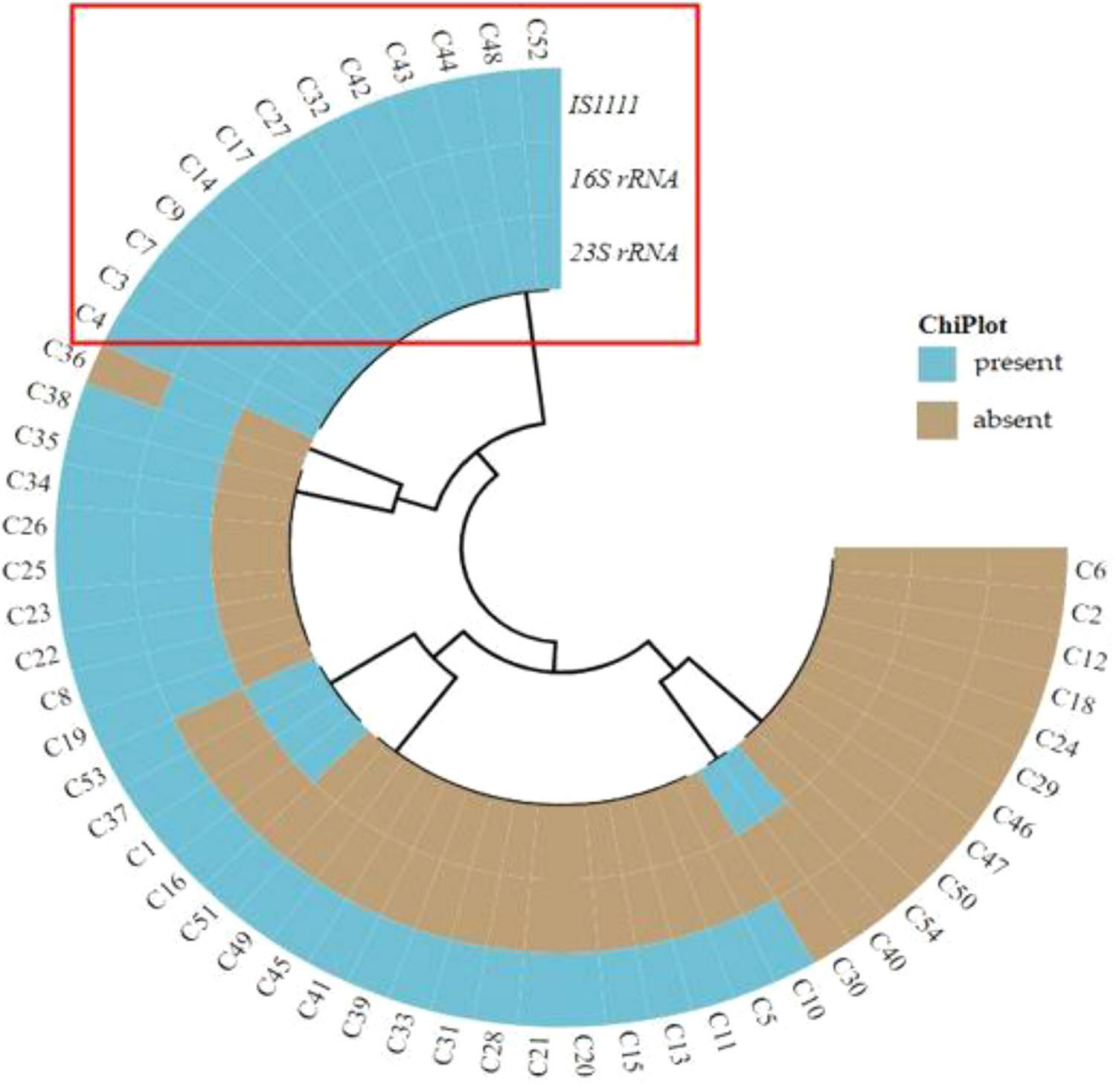
The clustering map showing the distribution of *23S rRNA*, *16S rRNA* and *IS111* genes for detection of *Coxiella burnetii* and *Coxiella* spp. in chickens. Samples within red box are confirmed as *C. burnetii*. The blue colour represents the presence of *23S rRNA*, *16S rRNA* and *IS111* genes, whereas brown is the absence of these three genes.

On a heatmap (Figure 2), a total of 17 PCR amplicon sequences from *Rattus* spp. for 3 tested genes (*IS111*, *23S rRNA* and *16S rRNA*) clustered together and were considered true *C. burnetii* positive infections, highlighted by the red box. Other *Coxiella* spp. were assigned to samples that tested positive for both the *23S* and *16S rRNA’*s but did not harbour the *IS111* gene. Therefore, these samples were not classified as *C. burnetii* and are annotated within a green box. A total of 13 PCR amplicon sequences from chickens for 3 genes (*IS111*, *23S rRNA* and *16S rRNA*) clustered together on a heatmap and were considered true *C. burnetii* positive infections as indicated within the red box (Figure 3).

## 4 DISCUSSION

As *C. burnetii* is the causative agent of Q‐fever, a global zoonosis, the detection of *C. burnetii* in rats and chickens is of particular significance (Filippitzi et al., 2017, Raele et al., 2018). The current study provides the occurrence of *C. burnetii* infections in two different hosts, 25% (17/68) and 24.1% (13/55) in *Rattus* spp. and chickens, respectively, by *IS111* markers. *Coxiella* spp. were also identified by *23S rRNA* and *16S rRNA* markers. Data from 2021 published by Mangena et al. (2021) carried out on cattle, sheep and pigs from the Free State Province reported an overall *C. burnetii* prevalence of 6.9%. Other studies have detected *C. burnetii* in ticks collected from dogs, cattle, goats, sheep, reptiles (Mofokeng et al., 2022; Mtshali et al., 2015, 2017; Wyk et al., 2022) and abattoir workers (De Boni et al., 2022) in South Africa. This study detected a total prevalence of 24.1% *C. burnetii* in chickens, which is higher than the prevalence reported in Japan but lower than in Iran, which were 4.2% and 17.2%, respectively, from egg samples using PCR (Rahimi & Doosti, 2012; Tatsumi et al., 2006). *C. burnetii* was detected in faecal samples from chickens and kidney tissues of rats in the current study indicating that pathogen is shed in faeces and/or urine.

The Q‐fever agent can infect wild pigeons (*Columba livia*), and some studies have linked foci of human and animal coxiellosis to pigeons (Babudieri & Moscovici, 1952; Ebani & Mancianti, 2022; Lang, 1990). Birds typically remove mites by preening, which exposes them to *C. burnetii* bacteria. *Dermanyssus gallinae* (the red poultry mite) can also encounter *Coxiellae* when using contaminated nesting materials like bird faeces (Ebani & Mancianti, 2022; Raele et al., 2018). Therefore, the detection of this pathogen in chicken faeces is not surprising, given that they can contract this bacterial infection from ingesting infected arthropod vectors or due to contact with contaminated faecal materials.

*Coxiella* spp. are exceptionally diverse and widespread in ticks but rarely detected in domestic animals (Seo et al., 2016). In this study, *Coxiella* spp. were detected in both rats and chicken samples by PCR using *23S rRNA* and *16S rRNA* markers. The percentage of positive samples in the current study was high in farms A (12.5%), D (11.7%) and C (10%), respectively. Elsewhere, other studies have used different PCR markers to detect *Coxiella*‐like endosymbionts (CLE) in ticks collected from horses in South Korea (Seo et al., 2016) ticks collected from different animals (cattle, dogs and goats) and the environment in Zambia (Kobayashi et al., 2021) and from ticks collected from bovines in India (Rialch et al., 2022).

Study of seroprevalence on *Coxiella* in human was conducted by De Boni et al. (2022), where they found the seroprevalence was 33% from abattoir workers from Free State and Northern Cape provinces of South Africa. Other studies reported the exposure of abattoir workers to *C. burnetii*, via inhalation of aerosols or dust contaminated with the bacteria (Eldin et al., 2017; Porter et al., 2011). From a public health perspective, the detection of *C. burnetii* in chickens confirms the pathogen's excretion in the environment and indicates that chickens and rats must be considered reservoirs in South Africa.

## 5 CONCLUSION

This study has used PCR and sequencing to detect the occurrence of *C. burnetii* and *Coxiella* spp. from wild rat tissue and chicken faecal samples. These wild rats roam around within chicken houses and surroundings and potentially provide a good opportunity for environment–rat–poultry interaction. Future studies should investigate the transmission cycle of *C. burnetii* and characterize other *Coxiella* spp. occurring in this rat–chicken environment. Ectoparasites such as mites and fleas could potentially infest rats and should be investigated as to whether they also harbour *Coxiella* spp. To the best of our knowledge, this is the first molecular detection of *C. burnetii* and *Coxiella* spp. from chicken and rat samples in South Africa.

## AUTHOR CONTRIBUTIONS

Tsepo Ramatla wrote the original draft manuscript and reviews. Oriel Thekisoe contributed to supervision, investigation, validation and methodology. Kgaugelo E. Lekota assisted in investigation and achieved experiments. Zamantungwa T. H. Khumalo and Asiashu Matshotshi contributed to methodology, achieving experiments. Moeti O. Taioe analysed data and reviews. All authors have read and agreed to the published version of the manuscript.

## CONFLICT OF INTEREST STATEMENT

The authors declare no conflict of interest.

## FUNDING INFORMATION

The authors did not receive any financial support from any university, company or institute.

### ETHICS STATEMENT

The Animal Research Ethics Committee approved this study following the criteria of the North‐West University Research Ethics Regulatory Committee (Ref No: NWU‐00274‐18‐A5).

### PEER REVIEW

The peer review history for this article is available at https://publons.com/publon/10.1002/vms3.1192.

## Data Availability

Raw data that support the finding of this study are available from the corresponding author, upon reasonable request.
